# Increased Risk of Central Serous Chorioretinopathy among Patients with Nonorganic Sleep Disturbance

**DOI:** 10.1155/2020/1712503

**Published:** 2020-02-12

**Authors:** Peng-Tai Tien, Chien-Yu Lai, Chun-Ju Lin, Wen-Lu Chen, Po-Kang Lin, Chih-Hsin Muo, Yi-Yu Tsai, Lei Wan, Wen-Chao Ho, Hui-Ju Lin

**Affiliations:** ^1^Graduate Institute of Clinical Medical Science, College of Medicine, China Medical University, Taichung, Taiwan; ^2^Department of Ophthalmology, China Medical University Hospital, Taichung, Taiwan; ^3^School of Chinese Medicine, College of Chinese Medicine, China Medical University, Taichung, Taiwan; ^4^School of Medicine, College of Medicine, China Medical University, Taichung, Taiwan; ^5^School of Medicine, National Yang-Ming University, Taipei, Taiwan; ^6^Department of Ophthalmology, Taipei Veterans General Hospital, Taipei, Taiwan; ^7^Management Office for Health Data, China Medical University Hospital, Taichung, Taiwan; ^8^College of Medicine, China Medical University, Taichung, Taiwan; ^9^Department of Biotechnology, Asia University, Taichung, Taiwan; ^10^Department of Obstetrics and Gynecology, China Medical University Hospital, Taichung, Taiwan; ^11^Department of Public Health, College of Public Health, China Medical University, Taichung, Taiwan

## Abstract

**Purpose:**

Patients with central serous chorioretinopathy (CSC) typically present with acute visual impairment and metamorphopsia. The disease previously has been associated with psychological stress. Population-based cohort studies on the risk of CSC among patients with nonorganic sleep disturbance (NOSD) are limited. An early sign of psychiatric disorder was probably sleep disturbance. Furthermore, psychological stress may be caused by sleep disturbance. We investigated the relationship between NOSD and the incidence of CSC.

**Design:**

Longitudinal cohort study. *Participants*. We used the Longitudinal Health Insurance Database and collected the data of 53,743 NOSD patients without CSC between 2000 and 2005 as the study group. Four-fold controls were selected randomly from those without neither sleep disturbance nor a CSC history with frequency matching of age, sex, and index-year.

**Methods:**

The difference in sex, age group, comorbidities, and steroid use between the two groups was analyzed by the *χ*^2^ test. Cox-proportional hazard regression was utilized to estimate the hazard ratio (HR) and 95% confidence intervals (95% CI) for comparison of the two groups. Kaplan–Meier analysis was applied to measure the cumulative incidence of CSC. Furthermore, the log-rank test was used to test the incidence difference between the two groups. *Main Outcome Measures*. The incidence rate of CSC in the following years until 2011 was detected.

**Results:**

During a mean follow-up of 7.36 ± 2.88 years, NOSD patients had a higher incidence of CSC than the controls (3.10 vs. 1.86 per 10,000 person-years; adjusted HR, 1.65; 95% CI, 1.34–2.02). Men had a higher risk of CSC than women. Sensitivity analyses stratified by sex, age group, or comorbidity condition showed consistently that NOSD patients had a higher risk of CSC than their controls. Dose-response showed that higher NOSD severity had even higher CSC risk.

**Conclusions:**

NOSD is an independent indicator for the increased risk of subsequent CSC development.

## 1. Introduction

Central serous chorioretinopathy (CSC) is a maculopathy typically presenting with relative central scotoma, metamorphopsia, and decreased central vision; it mainly affects middle-aged men [1, 2]. CSC is one of the most common nonsurgical retinopathies [2]. The typical finding of CSC is serous neurosensory retinal detachment over the posterior pole due to impaired activity of the retinal pigment epithelium and changes of choroidal hemodynamics [3, 4]. CSC is frequently self-limited with good recovering visual outcome [5]. However, CSC recurrence may result in considerable and irreversible visual acuity loss due to extensive retinal pigment epithelium damage [6].

The etiology of CSC remains unclear. The disease has been associated with psychological stress [7–9]. Studies also have revealed that endogenous hypercortisolism, psychopharmacologic medication, glucocorticoid medication, hypertension, elevated circulating cortisol and catecholamines, and peptic ulcer are risk factors for CSC [10–16]. Sleep disturbance is a common complaint and source of distress in community surveys of self-reported health problems [17–19]. An early sign of psychiatric disorder was probably sleep disturbance. Furthermore, psychological stress may be caused by sleep disturbance [20]. Leveque et al. [21] found CSC patients may be more likely than others to have sleep apnea. Kloos et al. [22] reported that sleep apnea may be a risk factor for the development of CSC. Bousquet et al. [8], Liu et al. [15], and Eom et al. [23] also reported that sleep disturbance was associated with CSC. Nevertheless, some studies revealed that patients with CSC were not statistically significant related to those with sleep apnea [24, 25]. The controversial results indicated that more and detailed studies are required to understand the association between sleep disturbance with CSC [15]. Since sleep apnea is generally with organic origin, in the present study, we intended to understand whether sleep disturbance with nonorganic origin exhibits higher risk of CSC. We analyzed and compared the incidences of CSC in the NOSD group and in the control group without NOSD using the national population-based dataset from Taiwan National Health Insurance (TNHI).

## 2. Methods

### 2.1. Database

The Taiwan Bureau of National Health Insurance (TBNHI) integrated all 13 public health insurance systems since 1995 into a large insurance program. Enrollment in this program is mandatory for people in Taiwan, and the covered rate is over 99% Taiwanese. TBNHI entrusted National Health Research Institutes to construct and maintain the National Health Insurance Research Database (NHIRD) from this program. The Longitudinal Health Insurance Database (LHID) was a part of NHIRDs, and it contained one million beneficiaries selected randomly from the year 2000 Registry of Beneficiaries. This database included all inpatient and outpatient medical claims for each beneficiary from the start of 1996 to the end of 2011. The International Classification of Diseases, Ninth Revision, Clinical Modification (ICD-9-CM) was used to identify the disease. The patient's identification code in the NHIRDs was recorded by TBNHI based on the Personal Information Protection Act. All researchers signed the written agreement for no intention of attempting to obtain patients' privacy. This study was also approved by the Research Ethics Committee in China Medical University Hospital, Taiwan.

### 2.2. Study Population, Outcome, and Comorbidity

We collected 53,862 patients with NOSD diagnosis (ICD-9-CM 307.4 and 780.5) and age at 20–50 years between 2000 and 2005 from LHID as the NOSD cohort. The date for NOSD diagnosis was defined as the index date. In the NOSD group, NOSD patients with CSC (ICD-9-CM 362.41) were excluded before the index date (*n* = 119). Controls were selected randomly from people in the LHID without sleep disurbance and CSC history as the control group, with frequency-matched criteria including age (5-year stratum), sex, and index-year at a ratio of 4 : 1.

All study subjects were followed from the index date to the date when CSC developed. All selected study subjects were followed to the date of withdrawal from this program or the end of 2011. The follow-up time, in person-years, was counted for each study subject. Potential comorbidity we considered included hypertension (ICD-9-CM 401–405), hyperlipidemia (ICD-9-CM 272), diabetes (ICD-9-CM 250), depression (ICD-9-CM 296.2, 296.3, 300.1, and 311), anxiety (ICD-9-CM 300.0, 300.2, 300.3, 308.3, and 309.81), alcohol-related disease (ICD-9-CM 291, 303, 305, 571.0, 571.1, 571.2, 571.3, 790.3, A215, and V11.3), smoking-related disease (ICD-9-CM 305.1, 430–438, 410–414, and 490–496), and obesity (ICD-9-CM). All comorbidities were defined before the index date. Patients who had received steroid treatment within 1 year before CSC development were defined as steroid users.

### 2.3. Statistical Analysis

The difference between the two groups, demographics, and comorbidity was compared by the *χ*^2^ test. The CSC incidences were measured in the NOSD and control groups. The hazard ratio (HR) and 95% confidence intervals (CIs) for CSC and CSC-associated risk factors were estimated in crude and adjusted Cox proportional hazard regression models. An adjusted model controlled for the variables that had significant differences in the crude model. Age-, sex-, comorbidity-, and steroid use-specific risks for CSC in the NOSD group were compared with those in the control group with the Cox model. Furthermore, we estimated the association between CSC and the severity of NOSD. The severity was classified as mild and serious according to NOSD patients who received sleeping pill treatment within 1 year before the CSC occurred. Kaplan–Meier analysis was used to measure the cumulative incidence for CSC during the study period in both groups, and the log-rank test was used to test the difference in incidences between both groups. SAS 9.4 statistical software (SAS Institute, Inc., Cary, NC, USA) was used for all statistical analyses in this study. The significant level was set at *P* < 0.05, 2-tailed test.

## 3. Results

We all collected 268,715 study subjects in this retrospective cohort study, 53,743 NOSD patients and 214,972 controls. In the NOSD group, there were more women than men (64.7% vs. 35.3%). The mean age was 36.8 ± 8.40 years. Compared with the controls, NOSD patients were more likely to have comorbidities, including hypertension (10.3% vs. 5.59%; 95%, *P* < 0.0001), diabetes (3.48% vs. 2.33%; 95%, *P* < 0.0001), hyperlipidemia (8.82% vs. 4.96%; 95%, *P* < 0.0001), depression (5.60% vs. 1.09%; 95%, *P* < 0.0001), anxiety (10.9% vs. 2.44%; 95%, *P* < 0.0001), alcohol-related disease (2.93% vs. 1.12%; 95%, *P* < 0.0001), smoking-related disease (25.4% vs. 13.9%; 95%, *P* < 0.0001), and obesity (1.22% vs. 0.77%; 95%, *P* < 0.0001). NOSD patients received steroid treatment more often than the control group (37.2% vs. 26.8%; 95%, *P* < 0.0001; Table 1).

**Figure 1:**
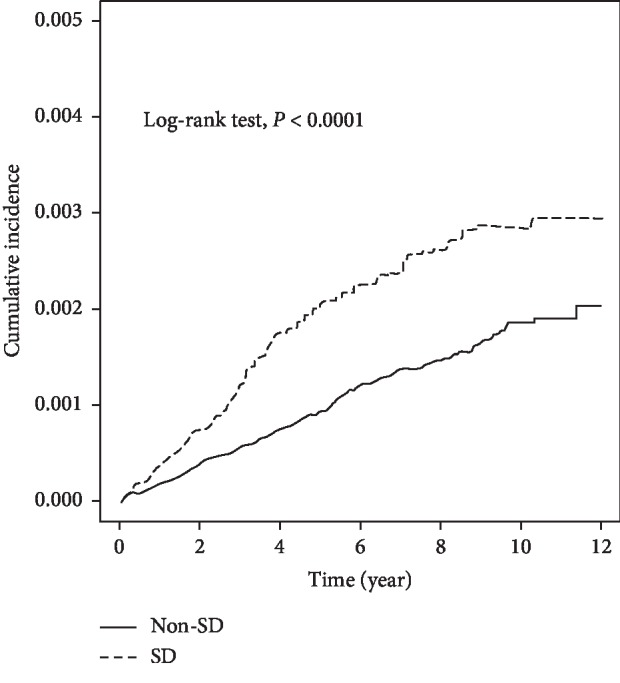
Cumulative incidence for central serous chorioretinopathy (CSC) in the no organic sleep disturbance (NOSD) and control cohorts. After 12 years of follow-up, the cumulative incidence for CSC in the NOSD cohort was approximately 0.09% higher than that in the control cohort (0.29% vs. 0.20%, log-rank test (*P* < 0.0001)).

During a mean 7.36 ± 2.88 years of follow-up, 281 and 144 patients had CSC in the control and NOSD groups, respectively, with incidences of 1.86 and 3.10 per 10,000 person-years, respectively (Table 2). After 12 years of follow-up, the cumulative incidence for CSC in the NOSD cohort was approximately 0.09% higher than the control cohort (0.29% vs. 0.20%, log rank test *P* < 0.0001; Figure 1).

NOSD patients had a 1.71-and 1.65-fold risk for CSC compared with non-NOSD controls in the crude and adjusted Cox models (95% CI, 1.40–2.09 and 1.34–2.02, respectively). In the multivariable Cox model, men and steroid usage had a higher risk for CSC than women and nonsteroid usage (HR, 2.51 and 1.33; 95% CI, 2.07–3.05 and 1.08–1.63, respectively; Table 2).

Table 3 presents the risk for CSC in the NOSD compared with the non-NOSD control group stratified by age, sex, comorbidity, and steroid use. NOSD women had a 1.72-fold higher risk than control women, and NOSD men also had a 1.59-fold higher risk than control men in the multivariable Cox model. No matter at what age, NOSD patients had a significantly more than 1.44-fold risk for CSC than controls. NOSD patients with or without any other comorbidity also had a higher risk of CSC. Furthermore, NOSD patients without steroid treatment had a significantly higher risk of developing CSC.

The association between CSC and NOSD severity is shown in Table 4. Of the NOSD patients, 61% (32,839/53,743) received sleeping pill treatment and were classified into the serious NOSD group. The CSC incidence increased with NOSD severity from 1.86 to 3.01 and 3.16 per 10,000 person-years in the controls, mild-NOSD, and serious-NOSD groups, respectively (*P* for trend < 0.0001). Compared with the control group, serious-NOSD patients exhibited 1.72-fold (95% CI, 1.35–2.20) increased risk of having CSC and a 1.55-fold risk (95% CI, 1.16–2.07) in the mild-NOSD patients.

## 4. Discussion

To our knowledge, this study is the first to assess the relationship of NOSD inducing CSC, with a large-scale nationwide representative database (*n* = 268,715) and long-term follow-up study design. NOSD patients had a higher risk for CSC, no matter in which sex, age group, or comorbidity condition.

NOSD patients had higher alcohol-related and smoking-related diseases. However, alcohol and smoking were not significant risk factors for CSC in our study. A recent study also had shown that tobacco was not involved in the development of CSC [15]. NOSD was related to CSC, independent of alcohol-related and smoking-related diseases. The severity of NOSD was defined by the use of sleeping pills. We found a higher incidence of CSC in patients with serious NOSD than mild NOSD patients, which suggested the importance of NOSD in the pathogenesis of CSC. The data also suggested that taking sleeping pills may increase the incidence of CSC. We did not analyze the effect of each different sleeping pills on the incidence of CSC in this study. We are now evaluating the outcomes of each different sleeping pills on the incidence of CSC.

It has been noted that corticosteroids were associated with the initiation, exacerbation or prolongation of central serous chorioretinopathy [11]. Another report indicated that not only the systematic but also inhalant or nasal use of steroid could increase the risk of CSC [15]. Nevertheless, NOSD patients had a higher CSC risk regardless of steroid use in our study, especially among those who did not undergo steroid treatment showing significant difference. In this study, we did not discuss the dosage and duration of the steroids, which may cause the discrepancy between our results and the reported data.

The significant points of our study are that the source of patients is based on a nationwide population-based database, which covers over 99% of the population in Taiwan and decreases the selection bias. Moreover, the longitudinal study was used to assess CSC risk-related NOSD.

This study has some potential limitations. First, diagnoses of CSC from health insurance database have been often challenged. However, the best effort had been made in this large population study based on NHIRD as in many reports in the literature [16, 25–28]. Second, personal information, such as psychological stress, alcohol/cigarette consumption, and body mass index (BMI), was not available from the administrative database, and this somehow may have compromised our results. However, after considering alcohol- and smoking-related diseases, the results in our study were not significant. Bordie et al. [24] proposed that BMI might bias the relationship between sleep apnea and CSC. Thus, we considered obesity as another comorbidity, and the result in our study was also not significant. Third, several asymptomatic or mildly symptomatic CSC patients may not have visited an ophthalmologic clinic, which may have led to underestimation of the risk with nondifferential misclassification. Moreover, there is not standardized diagnosis tool available on NOSD for each different physician. Some patients may actually suffer from different sleep disorders.

In our multivariable Cox model, men had a higher risk for CSC than women (HR, 2.51; 95% CI, 2.07–3.05; Table 2). This is consistent with a previous study showing CSC to be a male-predominant disease [1, 2, 26]. Sleep disturbance is a common health issue. Many sleep disorders, such as insomnia, hypersomnia, and sleep apnea, are included in sleep disturbance. Inadequate sleep is associated with cardiovascular disease [29]. CSC also was noted to be associated with an increased risk of stroke and coronary heart disease [27, 28]. Elevated catecholamine and cortisol levels were found in a sleep apnea population [30, 31]. Altered catecholamine levels and sympathetic activity often have been reported to accompany sleep disturbance [31, 32]. Sun et al. found that plasma concentrations of both catecholamines (epinephrine and norepinephrine) were significantly higher in active CSC than normal subjects, and then decreased to normal in the convalescent stage. They also found that plasma concentration of epinephrine was significantly correlated with macular edema. They proposed that prolonged stimulation of high catecholamine level might provoke focal RPE cells dysfunction, resulting in a breakdown of the outer blood-retinal barrier and then diffusion of fluids, which promoted CSC [33]. Those pathophysiological pathways might constitute a hypothetical connection between NOSD and CSC.

In conclusion, we demonstrated large-scale epidemiological evidence of a significantly increased risk for subsequent CSC development among an NOSD population. Decreased central vision or metamorphopsia in NOSD patients may need further macular examinations to exclude the possibility of CSC.

## Figures and Tables

**Table 1:** Demographic characteristics between NOSD and comparsion cohort.

Characteristics	NOSD *N* = 53743 (*n*)	%	Comparsions *N* = 214972 (*n*)	%	*P* value^$^
Sex					0.99
Women	34,774	64.7	139,096	64.7	
Men	18,969	35.3	75,876	35.3	
Age (years)					0.99
20–29	12,479	23.2	49,916	23.2	
30–39	18,627	34.7	74,508	34.7	
40–50	22,637	42.1	90,548	42.1	
Mean	36.8	8.40	37.1	8.25	
Comorbidity					
Hypertension	5548	10.3	12,026	5.59	<0.0001
Diabetes	1870	3.48	5000	2.33	<0.0001
Hyperlipidemia	4742	8.82	10,658	4.96	<0.0001
Depression	3010	5.60	2353	1.09	<0.0001
Anxiety	5876	10.9	5249	2.44	<0.0001
Alcohol-related disease	1577	2.93	2407	1.12	<0.0001
Smoking-related disease	13,645	25.4	29,896	13.9	<0.0001
Obesity	654	1.22	1646	0.77	<0.0001
Steroid users	19,964	37.2	57,563	26.8	<0.0001

^$^Chi-square test.NOSD: nonorganic sleep disturbance.

**Table 2:** Incidence and risk for CSC and associated comorbidities.

	Event no.	Person per years	Rate^§^	Crude HR^§^ (95% CI)	Adjusted HR^§^ (95% CI)
NOSD					
No	281	1,512,968	1.86	1.00	1.00
Yes	144	464,176	3.10	1.71 (1.40–2.09)^*∗∗∗*^	1.65 (1.34–2.02)^*∗∗∗*^
Sex					
Women	176	1,256,555	1.40	1.00	1.00
Men	249	720,590	3.46	2.47 (2.04–3.00)^*∗∗∗*^	2.51 (2.07–3.05)^*∗∗∗*^
Age (years)					
20–29	86	461,051	1.87	1.00	—
30–39	155	689,607	2.25	1.21 (0.93–1.58)	—
40–50	184	826,487	2.23	1.20 (0.93–1.55)	—
Comorbidity					
Hypertension					
No	387	1,849,898	2.09	1.00	1.00
Yes	38	127,246	2.99	1.44 (1.03–2.01)^*∗*^	1.08 (0.76–1.54)
Diabetes					
No	410	1,928,583	2.13	1.00	—
Yes	15	48,562	3.09	1.45 (0.87–2.43)	—
Hyperlipidemia					
No	386	1,866,904	2.07	1.00	1.00
Yes	39	110,241	3.54	1.72 (1.24–2.39)^*∗∗*^	1.34 (0.94–1.90)
Depression					
No	414	1,938,883	2.14	1.00	—
Yes	11	38,262	2.87	1.35 (0.74–2.46)	—
Anxiety					
No	401	1,897,576	2.11	1.00	—
Yes	24	79,568	3.02	1.43 (0.95–2.16)	—
Alcohol-related disease					
No	416	1,950,593	2.13	1.00	—
Yes	9	26,552	3.39	1.57 (0.81–3.04)	—
Smoking-related disease					
No	349	1,664,185	2.10	1.00	—
Yes	76	312,959	2.43	1.16 (0.91–1.49)	—
Obesity					
No	421	1961556	2.15	1.00	—
Yes	4	15589	2.57	1.18 (0.44–3.15)	—
Steroid usage					
No	279	1,420,146	1.96	1.00	1.00
Yes	146	556,999	2.62	1.34 (1.09–1.63)^*∗∗*^	1.33 (1.08–1.63)^*∗∗*^

^§^Per 10000 person-years. ^*∗*^*P* < 0.05, ^*∗∗*^*P* < 0.01, ^*∗∗∗*^*P* < 0.001. CSC: central serous chorioretinopathy, NOSD: nonorganic sleep disturbance, HR: hazard ratio, CI: confidence interval.

**Table 3:** Incidence and risk for CSC in NOSD compared with non-NOSD cohort stratified by age, sex, comorbidity, and steroid usage.

	NOSD event no.	Person per years	Rate^§^	Control event no.	Person per years	Rate^§^	HR 95% (CI) crude	Adjusted
Sex^1^								
Women	64	303,885	2.11	112	952,670	1.18	1.82 (1.34–2.48)^*∗∗∗*^	1.72 (1.26–2.35)^*∗∗∗*^
Men	80	160,291	4.99	169	560,300	3.02	1.69 (1.29–2.20)^*∗∗∗*^	1.59 (1.21–2.09)^*∗∗∗*^
Age (years)^2^								
20–29	34	105,361	3.23	52	355,690	1.46	2.24 (1.46–3.46)^*∗∗∗*^	2.26 (1.46–3.50)^*∗∗∗*^
30–39	52	161,231	3.23	103	528,375	1.95	1.69 (1.21–2.37)^*∗∗*^	1.62 (1.15–2.27)^*∗∗*^
40–50	58	197,584	2.94	126	628,903	2.00	1.50 (1.10–2.05)^*∗*^	1.44 (1.05–1.98)^*∗*^
Comorbidity^3^								
Without any one	68	254048	2.68	214	1190351	1.80	1.52 (1.15–1.99)^*∗∗*^	1.52 (1.15–2.00)^*∗∗*^
With any one	76	210128	3.62	67	322617	2.08	1.79 (1.28–2.48)^*∗∗∗*^	1.76 (1.26–2.44)^*∗∗∗*^
Steroid usage^4^								
No	92	292,090	3.15	187	1,128,056	1.66	1.94 (1.51–2.49)^*∗∗∗*^	1.90 (1.48–2.45)^*∗∗∗*^
Yes	52	172,087	3.02	94	384,912	2.44	1.26 (0.90–1.77)	1.28 (0.91–1.80)

^1^Adjusted for hyperlipidemia, hypertension, and steroid use. ^2^Adjusted for sex, hyperlipidemia, hypertension, and steroid use. ^3^Adjusted for sex and steroid use. ^4^Adjusted for sex, hyperlipidemia, and hypertension. ^§^Per 10000 person-years. ^*∗*^*P* < 0.05, ^*∗∗*^*P* < 0.01, ^*∗∗∗*^*P* < 0.001. CSC: central serous chorioretinopathy, NOSD: nonorganic sleep disturbance, HR: hazard ratio, CI: confidence interval.

**Table 4:** Incidence and risk for CSC among different NOSD severity.

	*N*	Event no.	Person-years	Rate^§^	Crude HR (95% CI)	Adjusted HR (95% CI)
Controls	214,972	281	1,512,968	1.86	1.00	1.00
NOSD						
Mild	20,904	56	185,833	3.01	1.67 (1.25–2.22)^*∗∗∗*^	1.55 (1.16–2.07)^*∗∗*^
Serious	32,839	88	278,343	3.16	1.73 (1.36–2.20)^*∗∗∗*^	1.72 (1.35–2.20)^*∗∗∗*^
*P* for trend				<0.0001	<0.0001	<0.0001

^§^Per 10000 person-years. Adjusted for sex, hyperlipidemia, hypertension, and steroid use. ^*∗*^*P* < 0.05, ^*∗∗*^*P* < 0.01, ^*∗∗∗*^*P* < 0.001. CSC: central serous chorioretinopathy, NOSD: nonorganic sleep disturbance, HR: hazard ratio, CI: confidence interval.

## Data Availability

The data used to support the findings of this study are included within the article.

## Abbreviations

CSC: Central serous chorioretinopathy
NOSD: Nonorganic sleep disturbance
HR: Hazard ratio
CI: Confidence intervals
TNHI: Taiwan National Health Insurance
TBNHI: Taiwan Bureau of National Health Insurance
NHIRD: National Health Insurance Research Database
LHID: Longitudinal Health Insurance Database
ICD-9-CM: International Classification of Diseases, Ninth Revision, Clinical Modification.
